# Awake venovenous extracorporeal membrane oxygenation and survival

**DOI:** 10.3389/fmed.2024.1394698

**Published:** 2024-04-24

**Authors:** Felix A. Rottmann, Viviane Zotzmann, Alexander Supady, Christian Noe, Tobias Wengenmayer, Dawid L. Staudacher

**Affiliations:** ^1^Department of Medicine IV - Nephrology and Primary Care, Faculty of Medicine and Medical Center, University of Freiburg, Freiburg, Germany; ^2^Interdisciplinary Medical Intensive Care, Faculty of Medicine and Medical Center, University of Freiburg, Freiburg, Germany; ^3^Department of Cardiology, Pneumology, Angiology, and Intensive Care, Ortenau Clinical Center Offenburg-Kehl, Freiburg, Germany

**Keywords:** extracorporeal membrane oxygenation, mobilization, acute respiratory distress syndrome, intensive & critical care, RASS, awake

## Abstract

**Objectives:**

Deep sedation on the ICU is linked to poor outcome. This study investigated the link between Richmond Agitation-Sedation Scale (RASS) and outcome in venovenous extracorporeal membrane oxygenation (V-V ECMO).

**Methods:**

We performed a secondary analysis of a single-center V-V ECMO cohort. RASS was used as a surrogate measure of sedation depth, patients with a score ≥ −1 were considered awake. V-V ECMO durations below 24 h were excluded. Primary endpoint was 30-day survival. Secondary endpoints were hospital survival and weaning from both ventilator and ECMO therapy.

**Results:**

A total of 343 patients were reanalyzed. The median age was 55 years and 52.2% (179/343) survived for 30 days after ECMO cannulation. Median duration of ECMO was 7.9 (4.7–15.0) days and the median duration of mechanical ventilation after ECMO cannulation was 11.8 (6.7–23.8) days.

In the whole cohort, median RASS on day one and seven after ECMO were − 4 (−4 to −1) and − 3 (−4 to 0), respectively. ECMO survivors consistently had significantly higher RASS scores during the first 7 days of ECMO compared to non-surviving patients (*p* < 0.01). On day two after ECMO, survival of awake patients (i.e., RASS ≥-1) was significantly better compared to sedated [i.e., RASS −4 to −2; OR 2.20 (1.28–3.71), *p* < 0.01] or unresponsive patients [i.e., RASS -5; OR 2.27 (1.15–4.64), *p* = 0.02]. The survival benefit of awake ECMO was consistent from day two to seven. Patients awake at least once during ECMO showed higher 30-day survival rates [64.4% vs. 39.6%, OR 2.75 (1.77–4.24), *p* < 0.01].

**Conclusion:**

In this retrospective study, awake patients on V-V ECMO showed higher 30-day survival rates compared to sedated or unresponsive patients. These data should encourage further research on awake V-V ECMO.

## Introduction

Deep sedation hampers mobilization (1). In our 10-year data on venovenous extracorporeal membrane oxygenation (V-V ECMO) support (2), mobilization was connected to improved outcome.

In V-V ECMO, mobilization seems safe and feasible (3–5). Mobilization however is only one of multiple ways how sedation impacts outcome. There are abundant data that light sedation on intensive care unit (ICU) is liked to outcome including reduction of delirium, distress, and enabling spontaneous breathing (6–9). In critically ill patients on ICU, including ECMO patients, data show that complications including bacterial pneumonia may be reduced in awake patients (10–13). Additionally, deep sedation related muscle loss is another typical complication, developing within days on the ICU (14–16).

We therefore hypothesized that awake V-V ECMO independently improves outcome. Here, we investigated the association of the Richmond Agitation-Sedation Scale (RASS) and 30-day survival.

## Methods

In this retrospective cohort study, we reanalyzed data on mobilization during V-V ECMO support (2). Primary endpoint was 30-day survival. Secondary endpoints were hospital survival and weaning from both ventilator and ECMO therapy. Inclusion criteria were an age of at least 18 years at cannulation, primary venovenous support (excluding veno-venoarterial and veno-arterial ECMO), and a duration of V-V ECMO support of at least 24 h. The ethics committee of the University of Freiburg (file number 21–1683) approved this registry.

**Figure 1:**
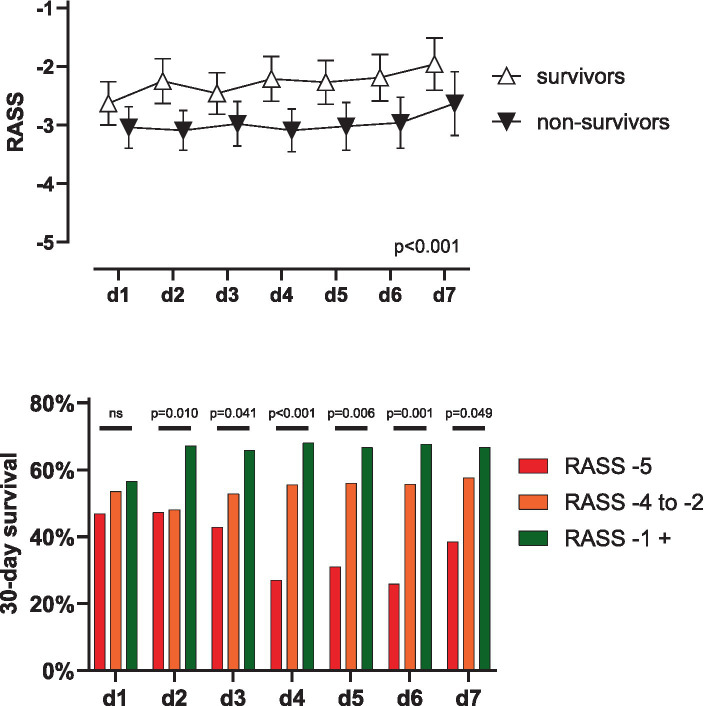
Outcome in V-V ECMO according to RASS-score. **(A)** Patients surviving 30 days showed significantly higher RASS-scores during day 1 to 7 (*p* < 0.001) while RASS-scores did not change over time and factors did not interact (*p* = 0.304 and 0.859). Data shown as mean with 95% CI. **(B)** On day two after ECMO-implantation, survival of awake patients (i.e., RASS-score ≥ −1) was significantly better compared to unresponsive patients [i.e., RASS-score − 5, 67.1 vs. 47.3%, OR 2.271 (1.145–4.638), *p* = 0.023] or sedated patients [i.e., RASS-score − 4 to −2, 67.1 vs. 48.1%, OR 2.200 (1.276–3.712), *p* = 0.004]. This trend was consistent to day seven. RASS, Richmond Agitation-Sedation Scale; *d*, day; ns, not significant; CI, confidence interval.

Daily decisions on sedation and mobilization are made individually at the bedside. For this analysis, we grouped patients into those with 30-day survival and non-survival. As for statistics, in Table 1, Mann–Whitney-U test was used on continuous data and Fishers Exact test on categorial data. In Figure 1A and Supplementary Figures 1A–3A, 2-way ANOVA was used to analyze the relation of RASS and survival. In Figure 1B and Supplementary Figures 1B–3B, Chi-square test was used to compare groups. Odds ratios were calculated using Fisher’s exact test. In Supplementary Table S1, univariate and multivariate logistic regression analysis was used based on predefined confounders of the primary endpoint. In Supplementary Table S2, 2-way ANOVA was used including only patients still on ECMO. Kaplan–Meier survival analysis was used for Supplementary Figure S4. A *p*-value of <0.05 was considered statistically significant. Data are given as median (interquartile range) or as number of patients (percentage of group).

**Table 1:** Patients characteristics and endpoints by 30-day survival under V-V ECMO.

Baseline characteristics	Total (*n* = 343)	Survivors (*n* = 179)	Non-survivors (*n* = 164)	*p*-value
Percentage of patients [%]	100	52.2	47.8	
Age	55 (45–64)	54 (42–61)	58 (46–67)	**0.001** ^**a**^
Female Gender	108 (31.5%)	55 (30.7%)	53 (32.3%)	0.816^b^
BMI	25.1 (23.6–30.2)	25.1 (23.5–31.3)	25.2 (23.6–29.2)	0.223^a^
**Preexisting conditions**				
Hypercholesterolemia	41 (12.0%)	20 (11.2%)	21 (12.8%)	0.740^b^
Nicotine use disorder	109 (31.8%)	58 (32.4%)	51 (31.1%)	0.817^b^
Coronary heart disease	37 (10.8%)	17 (9.5%)	20 (12.2%)	0.487^b^
Hypertension	124 (36.2%)	70 (39.1%)	54 (32.9%)	0.261^b^
Liver cirrhosis or chronic hepatitis	21 (6.1%)	6 (3.4%)	15 (9.1%)	**0.040** ^**b**^
Chronic kidney disease	23 (6.7%)	11 (6.1%)	12 (7.3%)	0.673^b^
Diabetes mellitus	49 (14.3%)	25 (14.0%)	24 (14.6%)	0.878^b^
Oncological disorders	59 (17.2%)	21 (11.7%)	38 (23.2%)	**0.006** ^**b**^
Immunodeficiency	93 (27.1%)	30 (16.8%)	63 (38.4%)	**<0.001** ^**b**^
Chronic lung disease	97 (28.3%)	41 (22.9%)	56 (34.1%)	**0.023** ^**b**^
CPR within 48 h before EMCO	32 (9.3%)	15 (8.4%)	17 (10.4%)	0.580^b^
**Respiratory status before ECMO**				
Horowitz index	70 (58–93)	71 (58–91)	69 (58–96)	0.824^a^
pO2 - arterial [mmHg]	65 (58–75)	64 (57–75)	65 (58–76)	0.263^a^
FiO2	1.0 (0.8–1.0)	1.0 (0.8–1.0)	1.0 (0.8–1.0)	0.122^a^
pCO2 - arterial [mmHg]	56 (46–73)	55 (44–68)	58 (47–76)	**0.033** ^**a**^
pH - arterial	7.3 (7.2–7.3)	7.3 (7.2–7.4)	7.2 (7.1–7.3)	**0.004** ^**a**^
Peak inspiratory pressure ≥ 42 cm H2O	33 (9.6%)	15 (8.4%)	18 (11.0%)	0.466^b^
**ICU stay**				
Duration of ICU stay from ECMO d1 [d]	13.7 (8.7–26.8)	21.9 (12.7–37.8)	9.5 (5.0–14.9)	**<0.001** ^**a**^
ECMO runtime [d]	7.9 (4.7–15.0)	7.9 (4.9–17.6)	7.9 (4.1–13.6)	0.052^a^
Mechanical ventilation [d]	11.8 (6.7–23.8)	16.8 (8.8–34.9)	9.4 (5.0–14.8)	**<0.001** ^**a**^
RASS day 1	–4 (–4 to –1)	–4 (–4 to –1)	–4 (–5 to –2)	
RASS day 2	–4 (–4 to –1)	–3 (–4 to 0)	–4 (–4 to –3)	
RASS day 3	–4 (–4 to –1)	–4 (–4 to –1)	–4 (–4 to –3)	
RASS day 4	–4 (–4 to –1)	–3 (–4 to 0)	–4 (–4 to –2)	
RASS day 5	–4 (–4 to –1)	–3 (–4 to 0)	–4 (–4 to –2)	
RASS day 6	–4 (–4 to –1)	–3 (–4 to 0)	–4 (–4 to –2)	See
RASS day 7	–3 (–4 to 0)	–3 (–4 to 0)	–4 (–4 to –1)	Figure 1A

Data given in median (interquartile range) or in number of patients (percentage of group).

*p*-values are calculated between groups using either ^a^Mann–Whitney-U test or ^b^Fishers Exact test.BMI, body-mass-index; CPR, cardiopulmonary resuscitation; ECMO, extracorporeal membrane oxygenation.

RASS was evaluated at least three times a day for each ECMO patient. For this analysis, the highest documented RASS score each day was considered. Patients were categorized as awake (RASS ≥-1), sedated (RASS −4, −3 or − 2) or unresponsive (RASS -5) according to highest RASS-score.

## Results

### Patient cohort

As previously reported (2), 343 patients were treated with V-V ECMO for ≥24 h between October 2010 and May 2021 (canulation window). 30-day survival was reached in 179/343 (52.2%) patients. Non-surviving patients suffered from significantly more preconditions, i.e., immunodeficiency, lung and liver disease and oncological disorders (all *p* < 0.05). There were no relevant differences in the respiratory status before ECMO cannulation, see Table 1. Surviving patients stayed significantly longer on the ICU after ECMO cannulation (21.9 (12.7–37.8) compared to 9.5 (5.0–14.9) days, *p* < 0.001) and were on mechanical ventilation significantly longer (16.8 (8.8–34.9) compared to 9.4 (5.0–14.8) days, *p* < 0.001). ECMO runtime was 7.8 (4.3–14.8) days not differing between the groups (*p* = 0.052).

### RASS on ECMO

Median RASS on the day of ECMO implantation was −4 (−4 to −1) and − 3 (−4 to 0) on day 7. A 2-way ANOVA showed significantly higher RASS-scores over the first 7 days in surviving patients (*p* < 0.001) while RASS-scores did not change over time (*p* = 0.304) without interaction (*p* = 0.859), see Table 1 and Figure 1A.

### Awake ECMO

When grouping patients’ consciousness, 30-day survival did not differ statistically on day one after ECMO (56.5 vs. 53.5 vs. 46.9%. *p* = 0.445) in awake, sedated and unresponsive patients, respectively. Starting on day two however, survival of awake patients was significantly higher compared to sedated (67.1 vs. 48.1%, OR 2.200 (1.276–3.712), *p* = 0.004) or unresponsive patients (67.1 vs. 47.3%, OR 2.271 (1.145–4.638), *p* = 0.023). This trend was consistent until day seven, see Figure 1B. Patients who were awake at least once during ECMO had a significantly higher survival rate (64.4% vs. 39.6%, OR 2.750 (1.773–4.240) *p* < 0.001). The outcome of awake patients on ECMO was also significantly better when evaluating the outcomes ‘hospital survival’, ‘weaning from ECMO’, or ‘weaning from the ventilator’, see supplementary Figures S1–S3. A Kaplan–Meier survival analysis also confirmed these findings, see Supplementary Figure S4.

### Bias

This retrospective registry study of awake ECMO faces a significant risk of bias. We performed a multivariate logistic regression analysis including potential reasons for cerebral damage including CPR before ECMO, which did not significantly correlate with our primary endpoint. Of note being awake during the first 7 days of ECMO was an independent predictor of the primary endpoint while mobilization was not, see Supplementary Table S1.

The 2-way ANOVA on RASS on ECMO showed similar results when only analyzing patients still on ECMO, see Supplementary Table S2.

## Discussion

The analysis showed significantly better survival in more awake patients, especially if patients survived until day two after ECMO cannulation.

There are many data showing that deep sedation correlates with poor outcome (17–23). Lighter sedation and daily interruption of sedation might influence outcome by various means one being better mobilization (13). Data from the ELSO registry showed better survival in patients with early mobilization and better mobilization among others in patients avoiding mechanical ventilation and thus sedation (11). These plausible results match smaller previous studies (24–27). We also showed this correlation of mobilization and outcome in this patient cohort (2). Mobilization however is not the only mechanism by which awake ECMO might influence survival (13) and mobilization was not an independent predictor of outcome in the logistic regression. The improved outcome of awake ECMO patients however was consistent over all investigates secondary endpoints. A potential confounder of these results might be that surviving patients have a higher chance of being awake at least once during ECMO compared to early deceased patients. This was addressed by excluding patients not surviving at least 24 h from the analysis and by focusing on the first 7 days of ECMO, only. When evaluating only patients still on ECMO (excluding patient weaned early from ECMO) our results could be confirmed.

Another important potential confounder is that sicker patients might have needed higher sedation depth. Low RASS therefore would be a marker of illness rather than independently influencing risk of death. This bias cannot be excluded and has to be considered when drawing clinical decisions from retrospective data.

We saw a significantly better survival in awake patients only on day two after ECMO implantation. Without stretching the data, this fact might suggest that the vulnerable first 24 h after ECMO do not have to be complicated by too ambitious sedation reduction.

Lastly, not all trials on awake patients on the ICU were positive (17). Reasons might be found in the heterogeneous patient cohort and the potential increased risk of accidental extubation (28) or ECMO decannulation (29–31) in awake patients.

## Conclusion

In this retrospective study, awake patients on V-V ECMO showed higher 30-day survival rates compared to sedated or unresponsive patients. There are many confounders and biases to be considered when interpreting retrospective data. Pending robust data, deep sedation strategies in V-V ECMO might be advisable only for specific indications.

## Data availability statement

The raw data supporting the conclusions of this article will be made available by the authors, without undue reservation.

## Ethics statement

The studies involving humans were approved by the Ethics committee of the University of Freiburg (file number 21–1683). The studies were conducted in accordance with the local legislation and institutional requirements. Written informed consent for participation was not required from the participants or the participants’ legal guardians/next of kin in accordance with the national legislation and institutional requirements.

## Author contributions

FR: Conceptualization, Data curation, Formal analysis, Investigation, Methodology, Validation, Visualization, Writing – original draft, Writing – review & editing. VZ: Conceptualization, Methodology, Validation, Writing – review & editing. AS: Conceptualization, Methodology, Resources, Supervision, Validation, Writing – review & editing. CN: Data curation, Investigation, Writing – review & editing. TW: Conceptualization, Methodology, Resources, Supervision, Validation, Writing – review & editing. DS: Conceptualization, Data curation, Formal analysis, Investigation, Methodology, Project administration, Resources, Supervision, Validation, Visualization, Writing – original draft, Writing – review & editing.
