# Supplementary material for: Awake venovenous extracorporeal membrane oxygenation and survival
Source: Front Med (Lausanne). 2024 Apr 24;11:1394698. doi: 10.3389/fmed.2024.1394698 (PMC11076729; doi:10.3389/fmed.2024.1394698)
Supplement: Supplementary file 1 [file Data_Sheet_1.docx]

# **Supplementary material**

|  | Univariate logistic regression analysis | | | Multivariate logistic regression analysis | | |
| --- | --- | --- | --- | --- | --- | --- |
| Variable | Hazard ratio | 95% confidence interval | p-value | Hazard ratio | 95% confidence interval | p-value |
| Female gender | 0.929 | 0.589 - 1.466 | 0.751 |  |  |  |
| Age | 0.977 | 0.962 - 0.992 | **0.003** | 0.976 | 0.960 - 0.992 | **0.003** |
| BMI | 1.022 | 0.997 - 1.048 | 0.086 |  |  |  |
| Preexisting pulmonary disease | 0.573 | 0.356 - 0.921 | **0.022** | 0.489 | 0.294 - 0.812 | **0.006** |
| Canulation mode (DL vs. SL) | 0.676 | 0.391 - 1.171 | 0.163 |  |  |  |
| CPR 48h to ECMO | 0.791 | 0.381 - 1.64 | 0.528 |  |  |  |
| Awake in first 7 days | 2.750 | 1.776 - 4.259 | **<0.001** | 2.973 | 1.887 - 4.684 | **<0.001** |
| ICU-MS ≥2 in first 7days | 1.159 | 0.526 - 2.554 | 0.715 |  |  |  |
| Mechanical ventilation [h] | 1.000 | 1.000 - 1.000 | 0.547 |  |  |  |

**Supplementary table 1: Binary logistic regression analysis on 30-day survival**

Predefined potential confounders for the primary endpoint (30-day survival) were tested in a univariate and multivariate logistic regression analysis. The p-value is reported in bold if the differences are statistically significant (p<0.05). Patients were considered awake when RASS-scores were ≥-1. Abbreviations: BMI: Body mass index, DL: dual lumen, SL: single lumen, CPR: Cardiopulmonary resuscitation, ECMO: extracorporeal membrane oxygenation, ICU-MS: Intensive care unit mobility score

| ICU stay | Total (n=343) | Survivors (n=179) | Non-survivors (n=164) |  |
| --- | --- | --- | --- | --- |
| RASS day 1 (n=343/179/164) | -4 (-4 to -1) | -4 (-4 to -1) | -4 (-5 to -2) |  |
| RASS day 2 (n=325/174/151) | -4 (-4 to -1) | -3 (-4 to 0) | -4 (-4 to -3) |  |
| RASS day 3 (n=302/162/140) | -4 (-4 to -1) | -4 (-4 to -1) | -4 (-4 to -3) |  |
| RASS day 4 (n=280/153/127) | -4 (-4 to -1) | -3 (-4 to 0) | -4 (-4 to -2) |  |
| RASS day 5 (n=249/132/117) | -4 (-4 to -1) | -3 (-4 to 0) | -4 (-4 to -2) |  |
| RASS day 6 (n=230/123/107) | -4 (-4 to -1) | -3 (-4 to -1) | -4 (-4 to -2) |  |
| RASS day 7 (n=199/108/91) | -3 (-4 to 0) | -3 (-4 to 0) | -4 (-4 to -1) |  |

**Supplementary table 2: 2-way ANOVA on RASS-scores and 30-day survival including only patients still on ECMO.** Data given as median (interquartile range). A 2-way ANOVA comparing RASS-scores only in patients still on V-V ECMO and excluding those already weaned from ECMO showed significantly higher RASS-scores in surviving patients (p<0.001) while RASS-scores did not change over time (p=0.270) and the two factors did not interact (p=0.913).

**Supplementary figures 1-3: Outcome in V-V ECMO according to RASS-score.**

**Figure 1 = hospital survival, 2 = ECMO weaning, 3 = ventilation weaning.** As: Patients surviving to hospital discharge (1A) or reaching successful ECMO- (2A) or ventilation weaning (3A) showed significantly higher RASS-scores during day 1 to 7 (all p<0.001) while RASS-scores did not change over time and factors did not interact (all p>0.05). Data shown as mean with 95% CI.

Bs: Higher RASS-scores on later days of the ICU stay corresponded with better hospital survival (2A) as well as ECMO- (2B) and ventilation-weaning (3B).

Abbreviations: RASS = Richmond Agitation-Sedation Scale; d = day; ns = not significant; CI = confidence interval

**Supplementary figure 4: Kaplan-Meier survival analysis.**

Awake patients (i.e. RASS ≥-1, blue line) showed significantly better rates of survival compared to sedated/comatose patients (i.e. RASS ≤-2, red line) in both the landmark analysis after day 1 (A) and after day 7 (B).

Abbreviations: ICU = Intensive care unit; V-V = venovenous; ECMO = extracorporeal membrane oxygenation
